# A Machine Learning-Based Computational Architecture for Unlocking Water Dynamics in Saturated Calcium Silicate Hydrate

**DOI:** 10.3390/ma19122631

**Published:** 2026-06-18

**Authors:** Chunlong Liu, Juntao Kang, Qimin Liu, Zechuan Yu

**Affiliations:** 1 School of Civil Engineering and Architecture, Wuhan University of Technology, Wuhan 430070, China; 348438@whut.edu.cn (C.L.); liuqm@whut.edu.cn (Q.L.); 2 Sanya Science and Education Innovation Park, Wuhan University of Technology, Sanya 572000, China

**Keywords:** calcium silicate hydrate, transport properties, high-throughput molecular dynamics, graph neural networks

## Abstract

The durability of reinforced concrete is closely related to the transport behavior of water and aggressive ions within the complex nanoporous network of calcium silicate hydrate. While molecular dynamics simulations provide critical atomistic insights into these confined transport behaviors, their immense computational cost limits their scalability to complex structural and temporal domains. To overcome this bottleneck, we propose a novel, modular computational framework that synergizes high-throughput molecular dynamics with advanced graph neural networks. By rigorously learning the mapping between the local atomic environment and kinetic behaviors, our model achieves high-fidelity predictions of pore water diffusion coefficients in saturated calcium silicate hydrate while improving computational efficiency by three orders of magnitude compared to conventional force field methods. Furthermore, the model demonstrates strong transferability and can accurately capture localized nonlinear diffusion characteristics in multiparticle pore structures with rough surfaces. Building on the interchangeability of this framework’s core modules, we envision a visionary multiscale computational strategy that dynamically couples nanoscale atomistic predictions with mesoscale simulations. This work not only provides an ultrafast, highly accurate tool for screening transport properties across diverse structural configurations but also lays the groundwork for next-generation multiscale modeling of chloride ingress, ultimately advancing the design of resilient and sustainable reinforced concrete.

## 1. Introduction

Calcium silicate hydrate (C-S-H), the predominant hydration product in Portland cement, constituting approximately 70% of the hydration products, is a critical component that imparts mechanical strength to cement and influences its durability [1]. C-S-H contains numerous nanoscale gel pores that serve as the primary pathways for water molecule transport [2]. Many mechanisms of durability deterioration in reinforced concrete, such as shrinkage cracking [3], freeze–thaw damage [4], and harmful ionic attack [5,6], are closely linked to water transport within these pores. Therefore, investigating water transport properties in C-S-H gel pores is fundamental for addressing durability issues of reinforced concrete.

Nuclear magnetic resonance (NMR) [7,8], quasielastic neutron scattering (QENS) [9,10], differential scanning calorimetry (DSC) [11,12], X-ray computed tomography (XTC) [13,14], and other experimental techniques have been employed to investigate the transport behavior of cement-based materials. Additionally, numerical models offer effective approaches to studying the transport behavior of porous materials [15,16,17,18]. Among them, the Lattice Boltzmann Method (LBM) is a numerical approach that can effectively describe fluid transport processes in porous media, and it has been applied to studies related to the transport properties of cementitious materials [19,20,21,22,23]. However, capturing interfacial effects and intermolecular interactions in C-S-H at the nanoscale, as well as predicting transport properties in nanopore-confined environments, is challenging when relying solely on experimental methods and numerical models.

Molecular dynamics (MD) simulations serve as a crucial component of the multiscale research method, offering valuable theoretical support and unique insights into the structural characteristics and physicochemical properties of C-S-H gels at the nanoscale [24,25,26,27,28,29]. Significant progress has been achieved in studying the transport properties of C-S-H gel at the nanoscale using the MD method. The MD simulation reveals that the nanopore space of C-S-H is hydrophilic. Strong interactions between water molecules and silicate pore walls restrict molecular movement [30]. Other studies further classify water molecules within the pore channels of C-S-H gel into three distinct categories: interlayer water exhibits the weakest diffusion, followed by surface-adsorbed water, while capillary water diffuses the fastest. Notably, interlayer water shows dynamics similar to those of a glassy state, primarily because the water molecules are constrained by a network of hydrogen bonds and ionic bonds [31]. In subsequent work, the team investigates the transport of water molecules and ions within conical nanoscale pores of C-S-H, and finds that ion transport rates are lower than those of water molecules. This transport disparity is attributed to ion adsorption, ion cluster formation, and the blocking effect exerted by the nanocone tip. An increase in the inclination angle further intensifies this blockage, thereby widening the discrepancy between water and ion transport rates [32]. Although MD plays an important role in studying the transport properties of C-S-H at the nanoscale, its computational cost increases significantly with the number of calculations and the simulation scale, thereby limiting its further application and development.

Machine learning (ML), with its unique ability to process complex datasets and identify structure-property relationships in materials [33,34,35,36], is emerging as a powerful tool to compensate for the limitations of traditional MD simulations [37,38,39]. Leveraging this capability, recent studies have integrated deep learning (DL) with MD to systematically investigate Na^+^ and Cl^−^ diffusion kinetics in C-S-H gel nanopores across varied ion concentrations and temperatures [40]. Studies on tobermorite have constructed effective ML potentials (MLPs) [41]. The results show that the MSD values predicted by MLPs align with those from ab initio MD simulations (AIMD) and increase computational efficiency by two to three orders of magnitude. Moreover, based on DL, enhanced sampling MD, and density functional theory (DFT), a high-precision potential is established to reveal the dissolution kinetic rates of Ca from water/dicalcium silicate interfaces under different temperature conditions [42]. The MLP developed in another study accurately predicts the structural characteristics and dynamic behavior of C-S-H and maintains computational accuracy in systems with different Ca/Si ratios [43].

Changes in the pore environment of C-S-H gels can affect the transport behavior of atoms within their pores. However, when investigating multiscale transport in C-S-H with complex and diverse structures, the computational cost of MD simulations becomes expensive. Existing efforts combining ML with MD have largely remained focused on the development of interatomic potentials, while lacking targeted strategies to reduce the computational cost associated with the diffusion coefficient, which is a key transport parameter. Meanwhile, current studies on the transport properties of C-S-H are still mainly limited to pore surfaces with smooth structures and single atomic configurations. Furthermore, conventional LBM simulates diffusion processes using the non-dynamic diffusion coefficient. This simplified approach overlooks microstructural details at the nanoscale and fails to capture nonlinear diffusion behaviors arising from dynamic changes in local conditions within complex environments. Critically, current multiscale models that integrate atomic-scale calculations with LBM experience information distortion and theoretical incompatibility. Therefore, establishing the correlation between atomic structure and micro-scale transport behavior remains a significant scientific challenge in this field.

This study develops a modular computational framework based on MD and ML. The framework comprises four workflows: parametric modeling, automated MD simulations, high-fidelity calculation of atomistic diffusion coefficients, and generation of predictive models. It also allows flexible replacement of four core modules, namely the target system, force field, equilibration procedure and neural network. Based on this modular framework, this study develops a predictive model for water diffusion coefficients that accounts for the effects of multiple parameters. Using the atomic structure of saturated C-S-H as input, the model directly predicts atomistic diffusion coefficients. It also achieves highly accurate prediction of local diffusion coefficients through averaging, while improving computational efficiency by three orders of magnitude compared with CSH-FF force field simulations. In addition, the predictive model shows strong generalizability in systems with rough pore wall structures. It thus provides a tool for the accurate and efficient evaluation of pore water diffusion coefficients within saturated C-S-H gel pores larger than 2 nm. Building on the modular design of this computational framework, this study further envisions a multiscale approach that dynamically links chloride-ion diffusion at the nanoscale with the evolution of ion concentration at the mesoscale. This approach provides a new strategy for refined simulation of ion diffusion in saturated cement hydration products, enhancing the ability to predict and mitigate the deterioration of reinforced concrete, and holds significant potential for extending structural service life and promoting sustainable development.

## 2. Modular Computational Framework

**Figure 1:**
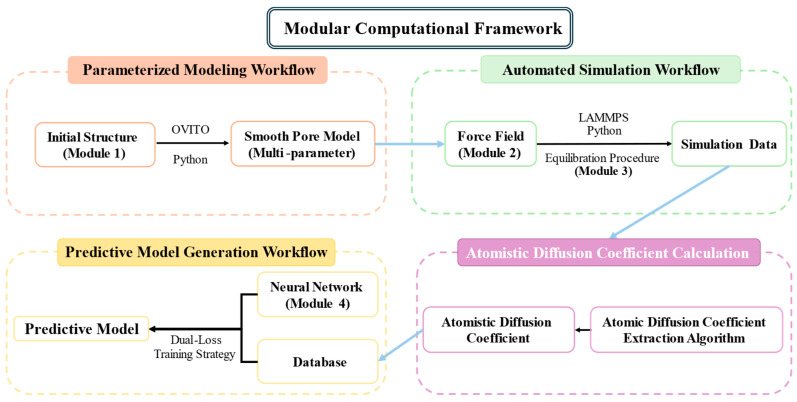
Schematic overview of the modular computational framework. Herein, the research object (Module 1), the simulation method (Module 2), equilibration procedure (Module 3) and the neural network (Module 4) can all be independently replaced or upgraded.

The scale and quality of training data are critical factors influencing the predictive accuracy and generalization capability of data-driven graph neural networks (GNNs). Traditional MD simulations typically employ single-parameter model construction or manual, one-off modeling approaches. Such inefficient workflows struggle to meet the extensive data requirements of GNNs for massive structural features and their corresponding dynamical properties. To address this need, designing a computational framework that integrates high-throughput generation with automated feature extraction becomes particularly crucial. Therefore, as shown in Figure 1, this study developed a modular computational architecture for developing predictive models of transport properties in cementitious materials (more details regarding this work are provided in the Supplementary Files).

### 2.1. Parameterized Construction of Smooth Pore Models

**Figure 2:**
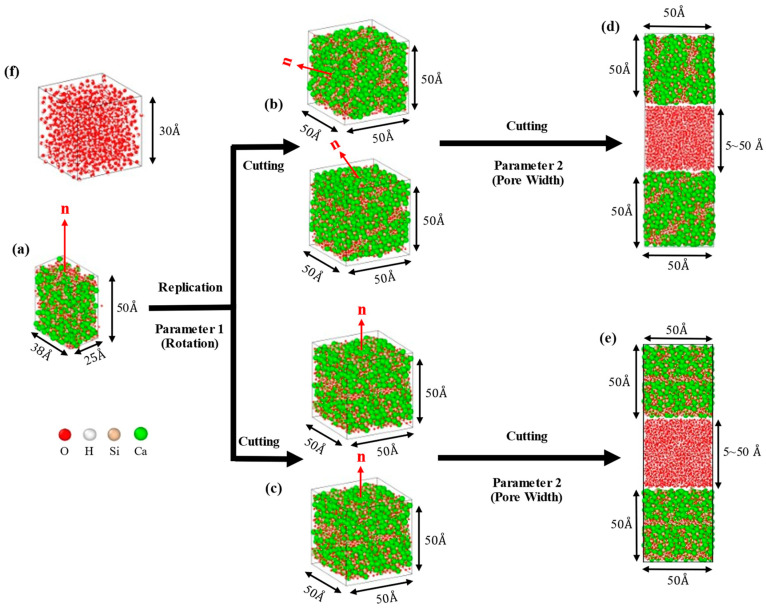
Parameterized construction workflow of the saturated smooth pore model. (**a**) Initial structure of C-S-H; (**b**) upper and lower C-S-H substrates with different atomic structures (rotated); (**c**) upper and lower C-S-H substrates with fixed atomic configurations (unrotated); (**d**) each model contains a unique atomic configuration of the C-S-H substrate, with pore widths uniformly sampled in the range of 5 Å to 50 Å; (**e**) all models adopt the same C-S-H substrate configuration, with pore widths varying from 5 Å to 50 Å at intervals of 5 Å; (**f**) bulk water model.

The parameterized construction of the smooth pore model is implemented by calling the OVITO (version 3.11.0) module in Python (version 3.12.4) to generate models with different atomic configurations and pore widths. As shown in Figure 2, the method is primarily governed by two core parameters: parameter 1 defines the rotational orientation of the C-S-H matrix using quaternions, while parameter 2 sets the pore widths. The construction of the initial model was based on the method proposed by Pellenq et al. [44], which has been thoroughly validated and widely adopted in the field. As shown in Figure 2a, the dimensions of the initial model are a = 38 Å, b = 25 Å, and c = 50 Å. The initial model has a density of 2.22 g/cm^3^, a Ca/Si ratio of 1.65, and a water mass content of 8.34%. This initial model is then replicated and extended along three orthogonal directions to form a 9 × 9 × 9 supercell. To introduce structural diversity in the C-S-H matrix, parameter 1 is employed to execute a random rotation on the constructed supercells. The precise value of this parameter is defined and quantified using quaternions. Subsequently, a C-S-H matrix of target size is extracted from the center region of the rotated or unrotated supercell along orthogonal coordinate axes. C-S-H matrices extracted from rotated and unrotated supercells are shown in Figure 2b and Figure 2c, respectively (the upper and lower C-S-H matrices in the same pore model are generated by applying different rotations to two supercells, followed by cutting). As shown in Figure 2d, the pore structure is generated by translating along the direction perpendicular to the (001) plane of the C-S-H matrix. Parameter 2 is used to precisely control the pore width. To construct the C-S-H pore model, it is also necessary to prepare pore water. A well-equilibrated bulk water box is duplicated and trimmed to obtain pore water with the desired width. The resulting pore water is then inserted into the target pore, thereby completing the construction of the pore model with three-dimensional periodic boundary conditions. By combining matrix structures generated through random rotations with randomly generated pore widths (ranging from 5 Å to 50 Å), a total of 128 saturated pore models were constructed. As shown in Figure 2e, to specifically investigate the effect of pore width, ten saturated pore models with defined pore widths (5 Å, 10 Å, …, 50 Å, at 5 Å intervals) are generated using unrotated C-S-H matrices. The 138 smooth pore models enable further investigation of how variations in the C-S-H matrix structure (resulting from different rotational orientations) affect the diffusion properties of saturated C-S-H, while maintaining a constant pore width. In addition, a saturated bulk water model (Figure 2f) is constructed to enable the neural network to learn the diffusion behavior of bulk water. Consequently, a total of 139 saturated smooth pore models are constructed for MD simulations in this study.

### 2.2. Automated MD Simulation Workflow

To improve the efficiency of high-throughput simulations, all MD simulations are carried out using Python scripts to drive LAMMPS (29 Aug 2024). The pore models are simulated in a three-dimensional orthogonal space with periodic boundary conditions, and the CSH-FF force field [45] is consistently used to describe interatomic interactions within the system. In the simulations, interparticle van der Waals interactions are modeled using a Lennard-Jones potential with an 8 Å cutoff radius. Electrostatic interactions are described by a Coulomb potential with a 10 Å cutoff radius. Long-range electrostatic interactions extending beyond this cutoff are treated using the particle-particle particle-mesh (PPPM) algorithm, with a specified precision of 10^−3^. The initialization procedure comprises four successive stages: an NVE ensemble simulation with a 0.01 fs time step for 1000 steps (10 fs) to eliminate atomic overlaps in the initial configuration; an NVT ensemble simulation with a 0.01 fs time step for 1000 steps (10 fs), during which the system is gradually heated from 50 K to 300 K; an NVT ensemble simulation with a 0.1 fs time step for 1000 steps (100 fs) to allow intermediate time step adaptation; an NVT ensemble simulation with a 1.0 fs time step for 1000 steps (1 ps) to complete the initial relaxation at the target temperature. The initialization phase concludes upon completion of these four stages. This progressive time step strategy is intentionally adopted to prevent energy drift or numerical instability during the thermalization of complex interfacial structures and represents a necessary stabilization measure.

Thereafter, the system is switched to the NPT ensemble (maintaining a temperature of 300 K and a pressure of 0 atm) and propagated for 1,000,000 steps (1 ns) with a fixed 1.0 fs time step. The first 0.5 ns is used for equilibration, and the final 0.5 ns constitutes the formal production run. During this entire simulation stage, structural configurations are recorded every 10 ps (10,000 steps). Concurrently, mean square displacement (*MSD*) values are output every 0.1 ps (100 steps). *MSD* analysis is performed on the 1 ns constant timestep trajectory, while the diffusion coefficient is calculated solely from the latter 0.5 ns of this constant timestep trajectory.

### 2.3. Atomic Diffusion Coefficient Extraction Algorithm

The relationship between the local environment of an atom and its diffusion behavior provides the data foundation for neural network models to effectively predict the diffusion performance of C-S-H under different atomic configurations. However, due to the nonlinear nature of individual atomic motion, the calculation of atomic diffusion coefficients remains difficult. The extraction of atomic-scale diffusion coefficients relies on atomic trajectory files and *MSD* data. Therefore, this study performs automated MD simulations on the 139 constructed models to obtain atomic trajectory files and *MSD* data. The formula for calculating *MSD* is given in Equation (1).

(1) $$MSD(t)=\frac{1}{N}\sum_{i=1}^{N}{\left\|r_{i}(t)-r_{i}(0)\right\|}^{2}$$

$r_{i}(t)$ represents the position of atom *i* at time *t*, while $r_{i}(0)$ represents the initial position of atom *i*. *N* denotes the total number of atoms.

A faster increase in *MSD* over time generally indicates stronger mobility. As shown in Equation (2), the slope can be obtained by fitting the *MSD* curve, and the diffusion coefficient can then be calculated. However, this process requires fitting over a sufficiently long time interval within the linear growth region after the system has reached equilibrium and based on a sufficiently large number of atoms.

(2) $$MDC=\frac{MSD(t+\Delta T)-MSD(t)}{6\Delta T}$$

In this equation, Δ*T* denotes the time interval, and *MDC* represents the mean diffusion coefficient of the system.

In the MD simulations in this study, each sample generates a 1 ns atomic trajectory, and atomic coordinates are output at a time interval of 10 ps. Therefore, each trajectory contains 100 frames of data. To reduce the influence of initial-configuration relaxation on the statistical results, the first 50 frames (0–0.5 ns) serve as the equilibration stage, and the remaining 50 frames (0.5–1.0 ns) serve as the post-equilibration production stage. This procedure helps ensure that the time evolution of the output *MSD* is more stable and statistically meaningful. Based on the *MSD* data obtained from the production stage, the *MSD*–*t* curve in the linear-growth region is fitted using Equation (2), and the mean diffusion coefficient of each atomic model is then calculated. The mean diffusion coefficient characterizes the long-time average diffusion behavior of all atoms in the equilibrated system (in this work, the time interval is set to 500 ps after equilibration). Meanwhile, for a system with a large number of particles, the long-time diffusion behavior tends to be a relatively stable value. In theory, since the diffusion coefficient derives from displacement statistics, the diffusion coefficient of an individual atom is further defined with reference to Equations (1) and (2). However, when the object of study narrows to a small atomic cluster or a single atom, directly applying the diffusion-coefficient formula introduces bias. For example, over longer time scales, an atom hops between different local environments, and its dynamical state changes with time, which violates the equilibrium assumption required to obtain an accurate diffusion coefficient. To address these issues, this study introduces the short-time displacement to characterize the relative mobility of atoms, and further employs mean displacement within a local region to rectify the quantification of atomic migration capability. The formula for calculating the short-time displacement is given in Equation (3), as follows:

(3) $$SQAD_{i}(\Delta t)=\|r_{i}(t+\Delta t)-r_{i}(t){\|}^{2}$$

Here, $r_{i}$ denotes the position of atom *i*, Δt denotes the time interval between two frames, and *SQAD_i_* represents the squared atomic displacement.

Subsequently, the squared short-time atomic displacement is further modified using Equation (4). The central atom and its neighboring atoms are selected to calculate the local squared atomic displacement, which can serve as an indicator for evaluating the relative mobility of the central atom.

(4) $$LSQAD_{i}=\frac{1}{M}\sum_{j=1}^{M}SQAD_{j}$$

*M* denotes the total number of atoms including the central atom *i* and its neighboring atoms, and *LSQAD_i_* represents the local average of the squared atomic displacement of atom *i* and its neighboring atoms.

This local statistical average effectively smooths out the short-term fluctuations of individual atoms. For example, the oscillatory motion of an atom: its back-and-forth movement causes positive and negative displacements to cancel each other out, resulting in a net displacement that approaches zero, even though the atom actually has high mobility. Although this local average value is not equivalent to the diffusion coefficient itself, it is directly proportional to the atomic diffusion coefficient. To convert the local average into the atomic diffusion coefficient, a calibration coefficient (*K*) is introduced here to recalibrate its diffusion performance. This coefficient represents the ratio of the system’s overall diffusion coefficient to the average of the local average values. Equation (5) for calculating *K* is as follows:

(5) $$K=MDC/\left(\frac{1}{N}\sum_{i=1}^{N}LSQAD_{i}\right)$$

*N* represents the total number of atoms in the C-S-H system, and *MDC* denotes the diffusion coefficient of the entire system.

Once the proportional coefficient *K* is obtained, the atomic-level diffusion coefficient can be calculated using Equation (6):

(6) $$ADC_{i}=K\cdot LSQAD_{i}$$

Given the complexity of C-S-H structures and the strong fluctuations in individual atomic dynamics, this extraction algorithm is not absolutely precise. Nevertheless, it remains capable of effectively evaluating the diffusion characteristics of individual atoms while remaining consistent with the long-time diffusion behavior of the system and satisfying physical constraints.

### 2.4. Generation of the Water Diffusion Coefficient Prediction Model

**Figure 3:**
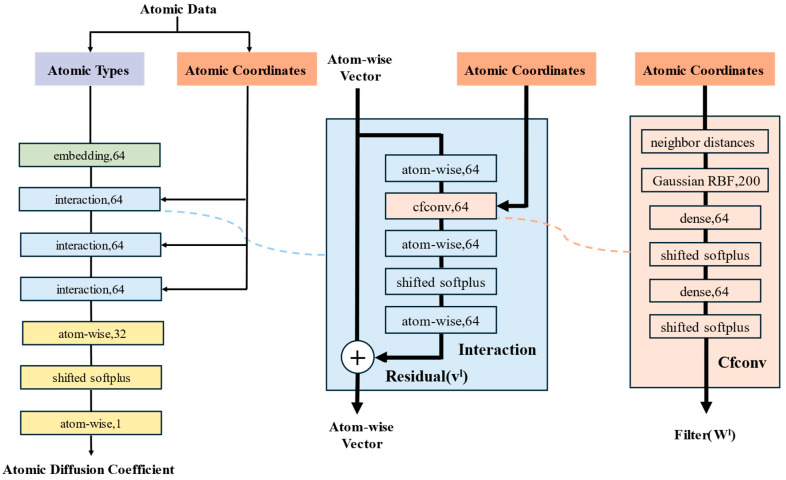
SchNet neural network architecture for predicting ADCs in saturated pore models. (**Left**) Overview of the SchNet architecture; (**Center**) detailed structure of the Interaction Block; (**Right**) continuous filter convolution layer and its associated filter generation network.

Figure 3 illustrates the architecture of SchNet [46,47], it is a continuous-filter convolutional neural network that can predict molecular energies and atomic forces by learning key features within molecular systems. This neural network rigorously incorporates fundamental physical constraints and effectively processes information regarding irregular spatial arrangements of atoms in molecular systems. Therefore, SchNet is implemented within the computational framework.

**Figure 4:**
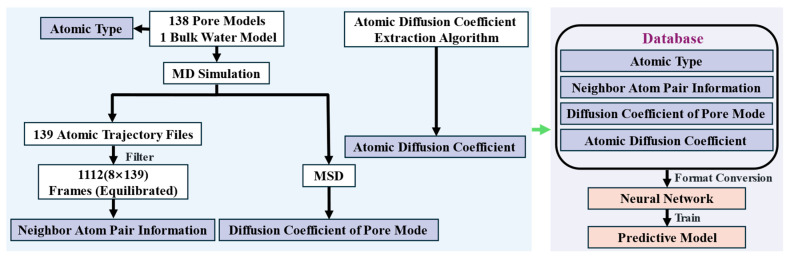
Workflow of Database Generation.

The construction quality of the database directly affects the fitting performance and generalization ability of the neural network model regarding the structure-property relationship. To ensure an adequate amount of data and structural diversity among the samples, selective sampling was performed on the equilibrated trajectories in this study. Specifically, 8 frames were extracted from each trajectory along the timeline at intervals of 5 frames, with the starting and ending frames set to 60 and 95, respectively. Consequently, data samples from 8 target frames were obtained for each pore model, thereby expanding the total number of samples to 1112. It should be noted that the samples extracted from the same trajectory are spaced by at least 50 ps, a duration sufficient for the atomic configurations to undergo significant changes. Therefore, such a sample augmentation strategy does not result in data leakage. As shown in Figure 4, this study subsequently utilized Python to comprehensively integrate the extracted atomic diffusion properties, the diffusion coefficients of each pore model fitted by *MSD* (encompassing both C-S-H and pore water), atomic types, and spatial configuration information, successfully constructing a structure-property database. SchNet takes atomic types and their local neighboring atom-pair information as inputs, thereby outputting atomic-level diffusion coefficients.

SchNet takes atom types and their local neighbor atom pair information as inputs to output the atomic-level diffusion coefficients. In this study, Mean Squared Error (MSE) is employed as the loss function, and two types of losses are introduced during the training process: atomic loss (loss1) and mean loss (loss2). The former evaluates the prediction accuracy of individual atomic diffusion coefficients, while the latter measures the model’s predictive capability for the overall diffusion behavior based on the statistical average of all atomic diffusion coefficients (both C-S-H and pore water) within the pore model. This joint optimization not only accounts for the accuracy of microscopic single-atom predictions but also statistically constrains the diffusion laws of the entire system, demonstrating the embedding of physical mechanisms into the neural network model. Given that atomic diffusion coefficients in different local environments can vary by several orders of magnitude, directly using raw diffusion coefficients as learning targets tends to weaken the model’s representation capability for low-to-medium value ranges. To mitigate this issue and simultaneously enhance the network’s sensitivity to relative errors, a logarithmic transformation is applied to the diffusion coefficients here. This transformation compresses the numerical range to a certain extent and suppresses the influence of extreme values, which is beneficial for improving the numerical stability of the training process and the overall fitting performance.

Considering the relevant settings of SchNet, the research object of this study, and the model’s prediction performance, the finally determined hyperparameters are shown in Table 1. Based on this configuration, 5-fold cross-validation is performed on the augmented dataset. Specifically, random seed 88 is used to control the data split, random seed 66 is used to control the random initialization of the model parameters, and random seed 99 is used to control the randomness during training; each fold is trained for 256 epochs.

## 3. Results and Discussion

### 3.1. Analysis and Validation of MD Simulation Results

**Figure 5:**
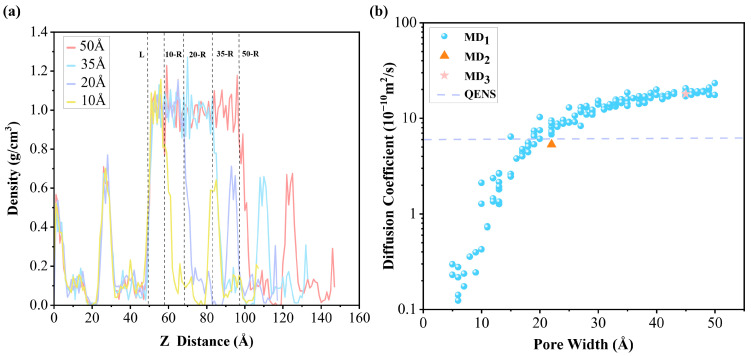
Effect of pore width on pore water density distribution and diffusion coefficient. (**a**) Water (including water molecules in both the substrate and the pore) density distribution within the pore model of the same initial C-S-H configuration (without rotation) at various initial pore widths (1.0, 2.0, 3.5, and 5.0 nm). L and R indicate the left and right boundaries of the interface between the C-S-H gel and pore water, respectively. (**b**) Relationship between the diffusion coefficient of pore water and the pore width. The y-axis is on a logarithmic scale. MD_1_ represents the simulation results obtained in this study. MD_2_ (2.2 nm) and MD_3_ (4.5 nm) denote simulation results from other studies. QENS represents the mean diffusion coefficient of pore water within mixed pore sizes below 100 Å (indicated by horizontal lines in the figure) as measured by QENS experiments.

The water molecule density distribution curve serves as an intuitive and effective tool for revealing the aggregation and spatial distribution of water molecules within C-S-H pore channels. Figure 5a demonstrates that the density distribution curves of pore water across various initial pore widths exhibit a consistent pattern. The water molecule density reaches a maximum value of approximately 1.2 g/cm^3^ near the C-S-H matrix boundary, reflecting the hydrophilicity of the C-S-H gel. As the distance from the interface increases toward the center of the pore channel, the water molecule density gradually decreases to approximately 1.0 g/cm^3^. This characteristic distribution of water molecule density can be attributed to the formation of hydrogen bonds between the hydrogen atoms of water molecules and the abundant oxygen sites present in the C-S-H matrix. In contrast, water molecules located farther from the C-S-H matrix exhibit a more homogeneous distribution due to the absence of direct interaction with the matrix [48]. The characteristics of these density distributions are in agreement with the results reported in a previous study [49].

The diffusion coefficient is an important physical index for assessing the kinetic properties of water molecules in C-S-H. As shown in Figure 5b, the diffusion coefficients of pore water obtained in this study agree with both previously reported simulation results [50,51] and experimental data [52]. The values remain of the same order of magnitude. Notably, the relationship between the equilibrated pore width and the diffusion coefficient of pore water is not a simple monotonic positive correlation. Some systems with larger pore widths exhibit lower pore water diffusion coefficients than those with smaller pore widths. However, this non-monotonic relationship between equilibrium pore width and pore water diffusion coefficient does not occur when the C-S-H matrix structure remains consistent. This is because the structural diversity of the C-S-H matrix leads to complex changes in atomic motion, ultimately affecting atomic transport properties (such as diffusion coefficients). Therefore, for structurally complex and diverse C-S-H systems, using traditional MD simulations to comprehensively assess the impact of structural diversity on the transport properties are often impractical because of the substantial computational resources required. In this context, DL techniques, which can efficiently process large and complex datasets, provide a more promising and applicable approach for studying such systems.

**Figure 6:**
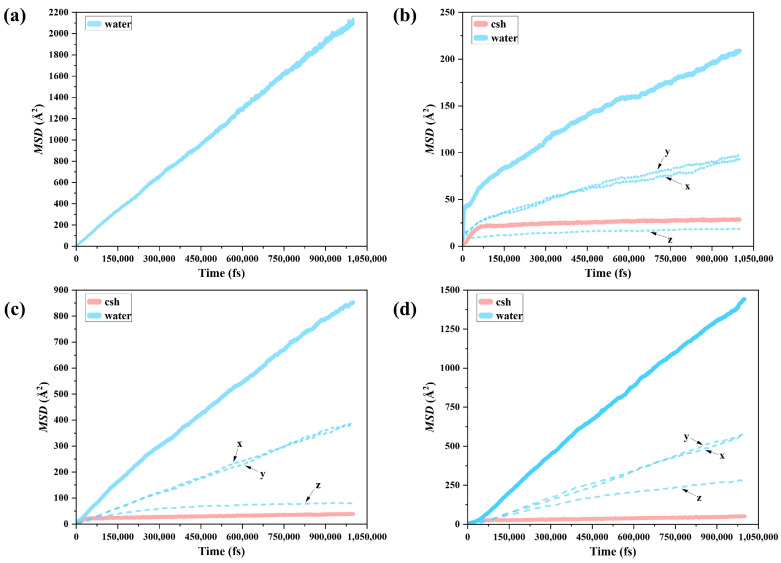
*MSD* evolution of pore water within the same (unrotated) initial C-S-H matrix under varying confinement conditions. (**a**) bulk water. (**b**–**d**) water confined within C-S-H pores of 1.0 nm, 2.5 nm, and 5.0 nm widths, respectively.

The mobility of atoms or ions within a system can be quantified by calculating the *MSD*. As shown in Figure 6, the *MSD* values of the C-S-H matrix remain largely unchanged with increasing initial pore width, exhibiting solid behavior. In contrast, the nanopore environment influences the kinetic behavior of pore water. As the pore width increases, the overall *MSD* of pore water increases monotonically and gradually approaches that of bulk water, as shown in Figure 6a. As shown in Figure 6b, at an initial pore width of 1 nm, the geometrical constraints of the narrow pores cause the *MSD* of water molecules along the z-direction (perpendicular to the C-S-H matrix plane) to be lower than that of the C-S-H matrix and of pore water in the x- and y-directions (parallel to the C-S-H matrix plane). Conversely, the *MSD* values of pore water in the x- and y-directions are essentially identical. Although pore water exhibits higher overall mobility than the C-S-H substrate, its motion is considerably restricted compared to bulk water. This inhibition arises from two primary factors: (i) interactions (hydrogen bonding, van der Waals forces, and Coulomb forces) between near-wall water molecules and the C-S-H substrate; and (ii) geometrical confinement by the nanopores in the z-direction. These effects restrict the free movement of water molecules. As shown in Figure 6c, the overall *MSD* of pore water increases when the initial pore width is raised to 2.5 nm. This phenomenon results from the influence of changes in pore geometry on the dynamic behavior of pore water. Increasing pore width elevates the proportion of water molecules with high diffusivity in the pore water system, thereby enhancing the overall mobility of pore water. Additionally, the *MSD* of pore water in the z-direction exceeds that of the C-S-H matrix, but remains lower than the values in the x- and y-directions. As shown in Figure 6d, the overall mobility of pore water further increases as the initial pore width is raised to 5 nm, more closely approximating the kinetic behavior of bulk water. Notably, pore water confined by the geometric structure of the C-S-H matrix consistently exhibits anisotropic diffusion distinct from that of bulk water. As the pore width increases, although the mobility of water molecules remains restricted by the geometric constraints of the C-S-H substrate, the *MSD*s in the x- and y-directions approach the corresponding bulk water values more rapidly, while the *MSD* in the z-direction remains consistently lower than those in the x- and y-directions. Consequently, the transport properties of pore water arise from the intricate coupling between its interactions with the C-S-H substrate and nanoscale geometrical confinement.

### 3.2. Assessment of Model Predictive Performance

#### 3.2.1. Stability Evaluation of the Predictive Model

**Figure 7:**
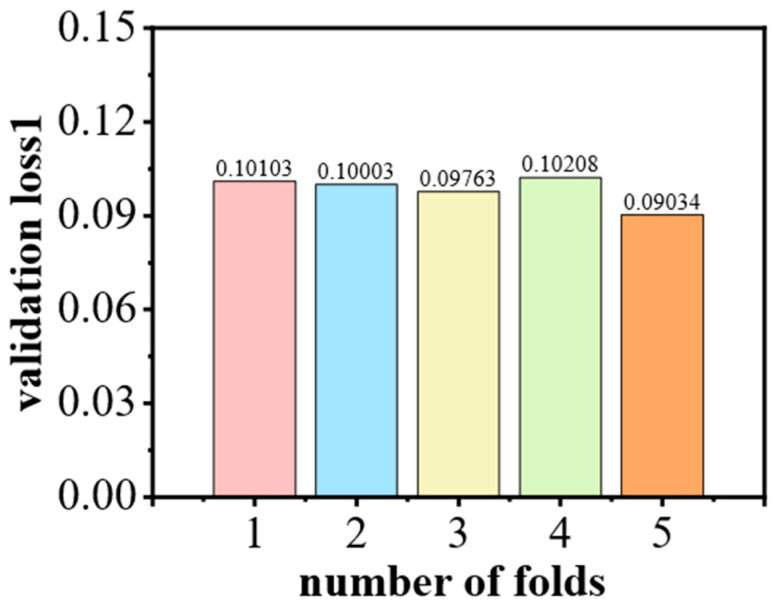
Variation in validation loss1 across the five folds of cross-validation.

This study will evaluate the performance of the predictive model in predicting the atomic-scale diffusion coefficient, the overall diffusion coefficient of pore water, and the local diffusion coefficient in pores. The predictive model can directly predict the atomic-scale diffusion coefficient, whereas the overall diffusion coefficient and the local diffusion coefficient are obtained by statistically averaging the atomic-scale diffusion coefficients. We use the validation-set loss1 as the selection criterion to identify the best-performing model in each fold, and we use the corresponding validation loss1 to assess model stability. As shown in Figure 7, the validation loss1 values across the five folds show minor variation, with a mean of 0.09822, a standard deviation of 0.00421, and a coefficient of variation of 4.28%. These results indicate that the model maintains stable performance across different datasets. Therefore, we select the model with median performance among the five folds to evaluate predictive accuracy and generalization performance.

#### 3.2.2. Training Convergence Analysis of the Prediction Model

**Figure 8:**
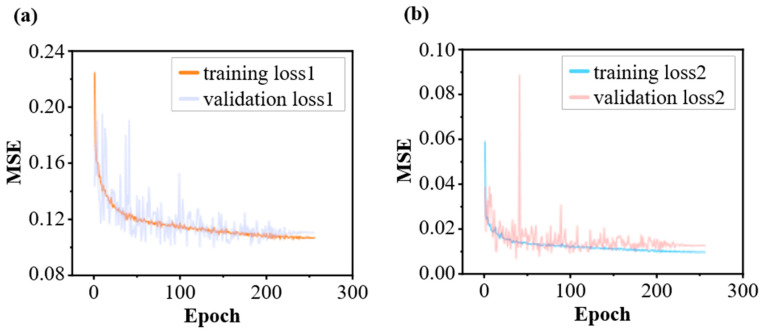
Variation in loss1 and loss2 with training epochs. (**a**) Variation in loss1 with training epochs; (**b**) variation in loss2 with training epochs.

The loss curves intuitively illustrate the variation in model loss during training, enabling the assessment of learning efficacy and the detection of underfitting or overfitting. Figure 8a and Figure 8b present the evolution of loss1 and loss2, respectively, throughout the training process. Both loss curves gradually decrease with training progression and learning rate decay until convergence, with no evidence of underfitting or overfitting. Compared with training loss, validation loss exhibits greater overall fluctuations. This arises because the training set directly participates in parameter optimization, resulting in tighter fitting and smoother loss curves. Notably, the diffusion behavior of individual atoms is more strongly influenced by variations in the local environment, which leads to a relatively large convergence value for loss1 (limited prediction accuracy). In contrast, the mean diffusion properties of a large number of atoms are more stable, and random prediction deviations caused by individual atoms can be effectively cancelled out upon local averaging. Consequently, loss2 demonstrates a smaller convergence value, and the mean prediction error of the predictive model will correspondingly be reduced.

#### 3.2.3. Accuracy Assessment of Atomic Diffusion Coefficient Prediction

**Figure 9:**
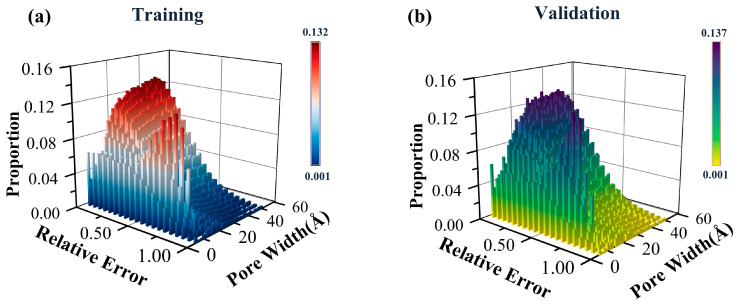
Distribution of the absolute relative errors of atomistic predictions under different pore sizes. Since the proportion of relative errors greater than 100% is small, these values are omitted from the figure. (**a**) Distribution of atomistic relative errors in the training set; (**b**) distribution of atomistic relative errors in the validation set.

Complex atomic dynamics, driven by the local environment, increase the difficulty of calculating and predicting atomistic diffusion coefficients. To analyze the distribution of prediction errors at the atomic scale and its variation with pore size, Figure 9 summarizes the fractions of atoms within different relative-error intervals for each pore width. The training set shown in Figure 9a contains approximately 5.91 million atomic samples, whereas the validation set shown in Figure 9b contains approximately 1.47 million. The error distributions in the two datasets show strong agreement, indicating that this pattern is not a chance phenomenon caused by a specific data subset, but rather a representative and statistically stable feature. The error distribution exhibits a transition near a pore width of 2 nm. When the pore width exceeds 2 nm, most atoms fall within intervals with relatively low absolute errors. As the pore width decreases below 2 nm, the relative-error distribution gradually extends toward higher-error intervals. This trend agrees with the findings reported by Hou et al. [53] and mainly arises from the complex solid–liquid interfacial effects under extremely narrow confinement below 2 nm. At a distance of approximately 1 nm from the C-S-H substrate surface, the dynamic behavior and structural characteristics of water molecules gradually transition from those of strongly confined interfacial water to those of bulk water. When the entire pore width is smaller than 2 nm, the interaction fields from the two opposing C-S-H surfaces become coupled within the narrow pore channel, making the force environment of the pore water molecules more complex. Under these conditions, the hydrogen-bond network and local orientational distribution of water molecules exhibit stronger structural ordering, and their diffusion behavior no longer follows a clear consistent trend but instead shows more complex nonlinear variations [54]. The inherently complex interactions under narrow confinement pose a significant challenge to the property prediction of neural networks. Therefore, this model is currently more suitable for saturated C-S-H systems with pore widths exceeding 2 nm. Although statistical averaging can effectively mitigate prediction deviations arising from individual atomic fluctuations and thereby improve the accuracy of mean-value predictions, it cannot fundamentally resolve the physical complexity under extreme confinement. In addition, the potential insufficiency of narrow pore configurations in the training set and the inherent limitations in the model’s representational capability also partially constrain its ability to resolve water diffusion behavior under extreme confinement. These factors collectively define the current applicability boundary of the model and indicate key directions for improving its predictive performance in the future.

#### 3.2.4. Accuracy Analysis of Global and Local Pore Water Diffusion Prediction

**Figure 10:**
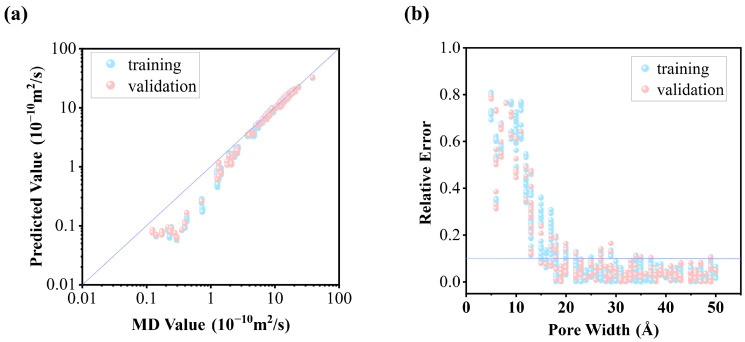
(**a**) Prediction accuracy of the diffusion coefficients of pore water as a function of pore size (the diagonal line represents x = y). (**b**) Comparison of the predicted diffusion coefficients of pore water with MD results (the horizontal line denotes a relative error of 0.1).

Figure 10a compares the predicted pore water diffusion coefficients with the MD simulation results. Although the dataset contains various complex pore configurations, the data points in the high-diffusion region remain closely clustered around the y = x line, indicating that the model can accurately capture diffusion behavior under different conditions and generalize well to the validation set. However, the model’s fitting performance in the low-diffusion-coefficient range is poorer than that in the high-diffusion-coefficient range, and this trend is consistent with the preceding atomistic accuracy analysis. Notably, the prediction accuracy of the mean diffusion coefficient is substantially higher than that of the atom-wise diffusion coefficients. This improvement suggests that statistical averaging reduces the diffusion uncertainty of individual atoms to some extent and thereby enhances predictive accuracy.

Figure 10b shows the distribution of the relative errors in pore-water diffusion predictions as a function of pore size. The error characteristics are highly consistent with the variation trends observed in the atom-wise diffusion-coefficient predictions. A separation in prediction error gradually emerges for samples with pore widths of around 2 nm. In extremely narrow confinements below 2 nm, the complex effects of coupled solid–liquid interfaces make accurate prediction challenging. For systems with pore widths greater than 2 nm, the relative prediction error remains mostly below 10%, and the mean relative errors on the training and validation sets are 0.035 and 0.042, respectively. The results indicate that this predictive model is applicable to predicting the diffusion coefficients of pore water in saturated C-S-H with pore sizes exceeding 2 nm.

**Figure 11:**
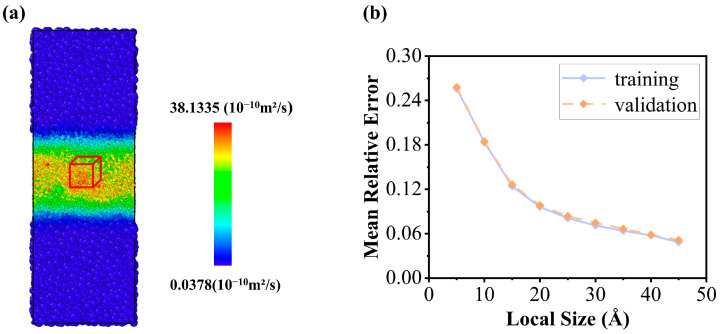
Predictive performance of local diffusion coefficients of pore water. (**a**) Schematic illustration of local pore water sampling, where different predicted atomistic diffusion coefficients are distinguished by color, and the red cube indicates the local region; (**b**) Variation trend in local diffusion coefficient prediction accuracy with local size.

Based on the local averaging of atomistic diffusion information, the predictive model estimates not only the average diffusion coefficient of the entire pore water system but also the local spatial diffusion characteristics of pore water. This local prediction capability provides a rapid means of analyzing local diffusion behavior within pores. As shown in Figure 11a, local predictions are performed for all samples with pore sizes greater than 2 nm, and the selected local regions inside the pores are defined as cubes. The cube edge length increases stepwise from 5 Å to 45 Å, and each selected region remains within the boundary of the corresponding pore water domain. For each edge length, 100 local regions are randomly sampled within the pore, and the mean relative error of the predicted diffusion coefficient is calculated to evaluate the effect of local scale on model performance. As shown in Figure 11b, the mean relative error of diffusion coefficient prediction decreases overall as the local sampling size increases, indicating that model accuracy continuously improves with increasing spatial averaging scale. When the local size reaches 4.5 nm, the mean relative errors on the training and validation sets decrease to 0.048 and 0.051, respectively, indicating that the model has high predictive accuracy. Previous analyses of pore water prediction show that, for pore sizes greater than 2 nm, the prediction accuracy does not vary significantly with pore size. This result suggests that once the pore size reaches approximately 2 nm, the number of atoms contained in pore water is sufficient for the error reduction effect of local averaging to approach saturation. Therefore, further increases in pore size provide only limited improvement in prediction accuracy, and the maximum accuracy gain attainable through local averaging is already nearly reached, if not exceeded. In addition to prediction accuracy, the model also shows advantages in computational efficiency. Under identical computational configurations (64 core CPU, 125.6 GB RAM, and NVIDIA GeForce RTX 3090 GPU), the trained model achieves prediction speeds approximately three orders of magnitude faster than CSH-FF force field calculations for systems comprising approximately 30,000 atoms. This result indicates that the water diffusion coefficient prediction model developed in this study substantially reduces computational cost and significantly improves the efficiency of high-throughput screening. Because the model can predict atomistic diffusion coefficients and benefits from the accuracy improvement provided by local averaging, it shows strong potential for application to larger systems and more complex structures.

### 3.3. Predictive Model Transferability Validation

#### 3.3.1. Construction Procedure for the Rough Pore Model

**Figure 12:**
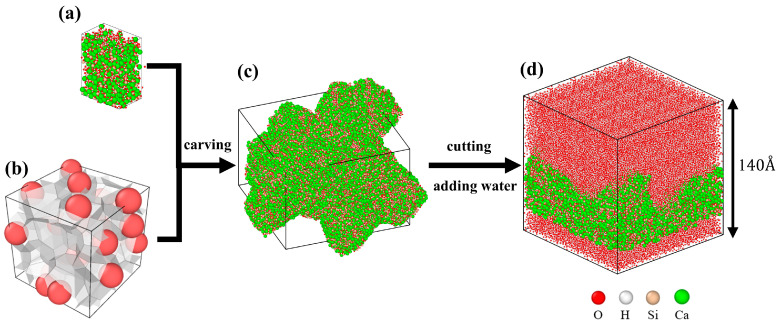
Generation procedure of the rough pore model. (**a**) Initial C-S-H structure; (**b**) Voronoi analysis; (**c**) C-S-H model with multi-particle features; (**d**) rough pore model.

To further investigate the upper limit of local-average prediction accuracy as the local scale or number of atoms increases, and to evaluate the generalization ability of the developed predictive model in larger and more complex saturated C-S-H systems, this study constructs a saturated rough-pore model based on the remapping method proposed by Yu et al. [28]. As shown in Figure 12a–c, the method starts from the spatial distribution of coarse-grained (CG) particles and performs a Voronoi tessellation in three-dimensional space. It then uses the initial C-S-H structure generated by the method of Pellenq et al. to build a sufficiently large C-S-H supercell. The supercell is then rotated according to the orientation information of the CG beads. Geometric trimming is subsequently applied to shape the supercell into a form consistent with the Voronoi polyhedra, thereby producing a C-S-H structural model with a multi-particle morphology. As shown in Figure 12d, geometric cutting is applied to the bottom and lateral sides of the model to obtain relatively flat C-S-H surfaces, while the top side retains the original rough interface formed by the packing of multiple particles. Water molecules are then introduced at the upper and lower surfaces, and periodic boundary conditions are applied in the three directions to construct a pore with a rough structure. The model has dimensions of 140 Å in all three spatial directions and contains a total of 260,000 atoms. In real materials, transport processes are jointly governed by spatial confinement effects and complex interfacial interactions. Compared with the idealized smooth-walled pore model described earlier, the rough pore walls formed naturally by particle packing make the migration behavior of water molecules within the pore more representative of the transport process in real materials. Therefore, this rough-pore model provides a structural basis for subsequent evaluation of the upper atom-number limit for local averaging and of the predictive performance of the neural network model for larger C-S-H systems with more complex pore structures.

#### 3.3.2. Evaluation of Predictive Model Transferability

**Figure 13:**
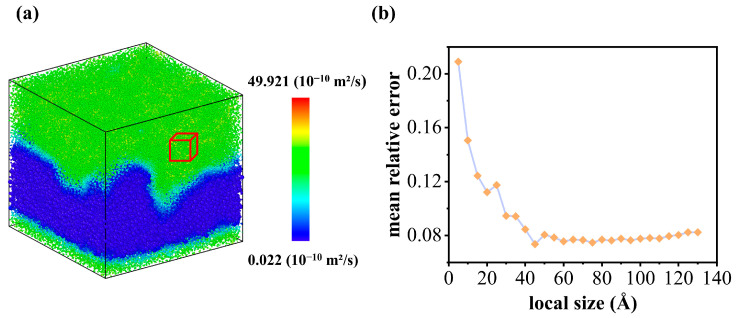
Prediction performance for local pore water diffusion coefficients in the rough pore model. (**a**) Schematic illustration of random local prediction, where different predicted atomistic diffusion coefficients are distinguished by color, and the red cube denotes the local region. (**b**) Mean relative error of local prediction as a function of local window size.

As shown in Figure 13a, the side length of the local subdomain starts at 5 Å and increases to 130 Å in 5 Å increments. To ensure a statistically meaningful evaluation, 100 random samples are generated at each local scale. The mean relative error serves as the metric for local prediction accuracy. As shown in Figure 13b, in the saturated C-S-H system with a side length of 140 Å, the neural-network prediction error generally decreases monotonically and gradually converges as the local domain size increases. The error reaches its minimum value of 0.0736 at a local domain size of 45 Å. This size therefore represents the optimal local prediction scale for the model. From the perspective of computational cost, the network serves as a low-cost predictive model for diffusion in nanoscale saturated C-S-H systems. Compared with force-field-based MD simulations, it reduces computational time by approximately three to four orders of magnitude. It therefore provides a feasible route for the rapid evaluation of transport properties in large-scale C-S-H systems with complex pore structures. This study verifies the feasibility of the modular computational framework from four perspectives, namely model stability, prediction accuracy, computational efficiency, and generalization ability, and lays the foundation for its future application to the prediction of chloride-ion diffusion in C-S-H pores.

### 3.4. Multiscale Computational Concept

**Figure 14:**
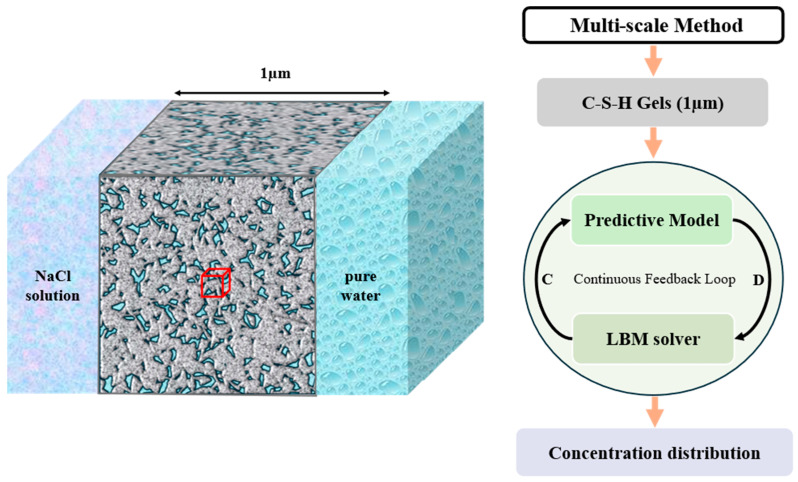
Multiscale Simulation Workflow for Cl^−^ Diffusion (The red cubes denote the lattice used for LBM). C denotes the Cl^−^ concentration distribution. D represents the Cl^−^ diffusion coefficient.

In classical LBM, the diffusion coefficient is typically treated as a constant throughout the entire computational domain. Although this rigid treatment simplifies the calculation process, it fails to accurately capture the nonlinear diffusion characteristics of atoms resulting from changes in the local environment, thereby limiting simulation accuracy. As illustrated in Figure 14, this study presents a multiscale computational concept featuring a bidirectional updating mechanism. In this multiscale computational concept, the diffusion coefficient is no longer treated as a fixed input parameter; instead, it serves as a dynamic parameter that is calculated and updated in real time throughout the simulation process.

The modular design of the computational framework allows replacement of the prediction target, force field, and neural network modules, thereby enabling construction of a new Cl^−^ structure-diffusion property database and training of a predictive model for Cl^−^ diffusion coefficients. Taking the diffusion of Cl^−^ within a saturated C-S-H cube with a side length of 1 μm as a representative example, the C-S-H matrix is discretized into multiple lattice cells (represented by the red cubes in the schematic), and a discrete velocity model is applied to simulate the spatial-temporal evolution of Cl^−^ diffusion. The left face of the cube is designated as an ion source with a prescribed concentration, while the right face is set as a zero-concentration sink; the remaining four faces are treated as impermeable, zero-flux boundaries.

During the computational process, the global atomic configuration of the C-S-H at the micrometer scale is imported into the predictive model to determine the diffusion coefficient of each individual atom. Through local statistical averaging, the effective diffusion coefficient for each lattice cell is obtained. Based on this predicted diffusivity distribution, the LBM solver executes collision and propagation operations across all lattice cells to update the concentration distribution throughout the C-S-H matrix. Subsequently, the newly generated three-dimensional concentration field is not directly used as the input for the LBM computation in the next time step; instead, it is immediately fed into the predictive model to regenerate a new diffusion coefficient distribution. The LBM solver then employs these real-time updated diffusion coefficients for the next round of concentration calculations, and this dynamic updating process continues iteratively until the system reaches a steady state.

This study envisions a multiscale computational concept that integrates the atomistic-scale material structure–property relationships calculated by the predictive model with the micrometer-scale transport behavior simulated by the LBM, thereby offering the potential for a more refined simulation of diffusion processes driven by Cl^−^ concentration gradients in saturated C-S-H gels.

### 3.5. Limitations and Prospects

The framework proposed in this study is applicable to the investigation of transport properties within saturated C-S-H gels. In the unsaturated state of C-S-H gel pores, transport mechanisms show complexity and nonlinearity. For example, within specific saturation ranges, water bridges may form at the pore. This phenomenon creates effective percolation pathways, which can enhance diffusion beyond that of both lower saturation and fully saturated pore water states. However, accurately defining the saturation range that enhances diffusion and characterizing the diverse and dynamic water distribution within pores require extensive MD simulations. This complexity and substantial data requirements present significant challenges to training a predictive model with generalization capabilities across diverse unsaturated systems. Unsaturated transport, which more closely reflects the actual transport conditions in C-S-H, should be a key focus in future research.

To overcome the predictive bottleneck under strong nanoconfinement, future research could further expand the training dataset through the targeted inclusion of additional narrow-pore C-S-H configurations and integrate neural network architecture optimization or more advanced deep learning models, thereby significantly enhancing the prediction accuracy of diffusion coefficients in the strongly confined regime.

The present work is fundamentally a proof-of-concept study designed to validate the feasibility of the proposed computational framework. While the current dataset adequately supports the conclusions drawn herein, the framework’s validation has been conducted under a fixed modeling parameter, structure generation method, force field, and simulation workflow, which imposes certain limitations. Leveraging the modular architecture of the framework, future investigations can systematically evaluate its transferability under varying computational conditions. It should be noted that directly applying the predictive model developed herein to structural data generated by alternative simulation protocols may introduce minor deviations. However, such discrepancies can be effectively mitigated by fine-tuning the existing model with a targeted set of newly generated simulation data, thereby enabling transferability and compatibility across diverse computational workflows.

For the prediction of reinforced concrete durability, this modular design enables the future development of highly accurate Cl^−^ diffusion prediction tools applicable to various cementitious materials and a wide range of service conditions. Therefore, it is necessary in future work to construct Cl^−^ diffusion models that incorporate more diverse atomic configurations, such as different assemblages of hydration products. Meanwhile, the simulation environment should be gradually extended to more complex scenarios, such as incorporating the effects of temperature gradients on transport properties. To enhance data reliability, high-precision force fields that are better suited to describing ion transport in cement hydration products should be selected. In addition, the validity of all simulation results needs to be rigorously established through comparison with experimental data. Further improvement of the algorithm is required. The performance of advanced models such as MACE [55], Allegro [56], and Neural Equivariant Interatomic Potentials (NequIP) [57] can be benchmarked and compared using optimized training strategies, allowing for the selection of an architecture that achieves balanced performance in terms of predictive accuracy, computational efficiency, and physical consistency. Through the multidimensional optimization described above, it is expected that a high-performance predictive model can be developed, thereby providing more robust and efficient computational support for multiscale iterative simulations of Cl^−^ diffusion in reinforced concrete. In the future, this conceptual multiscale approach will require further implementation and refinement in many specific details. However, the current proposal of this multi-scale method has contributed to more accurate prediction of the corrosion initiation time of reinforced concrete in marine environments, as well as elucidating the influence of nanoscale pore structures on Cl^−^ migration pathways.

## 4. Conclusions

To reduce the high computational cost required for simulating the transport properties of C-S-H gel, this study develops a modular computational framework with four workflows: programmatic construction of multiparameter models, automated high-throughput MD simulations, calculation of atomistic diffusion coefficients, and generation of predictive models. The predictive model developed within this framework efficiently predicts the diffusion behavior of water molecules in C-S-H nanopores. It therefore demonstrates the feasibility of the modular computational framework and serves as an efficient tool for exploring the relationship between complex C-S-H structures and their transport behavior. The main conclusions of this study are as follows:

(1) This study elucidates the diffusion dynamics of pore water under different controlling variables. Using the developed modular computational framework, this study constructs smooth pore models with different pore widths and diverse atomic configurations and performs automated simulations of water diffusion. The simulation results show that the density distribution and dynamic behavior of pore water are jointly governed by C-S-H interfacial interactions and pore size. These results effectively confirm the accuracy of the simulation data.
(2) This study defines the applicable spatial-scale boundary of the predictive model. The performance evaluation reveals a clear spatial-scale effect on the model accuracy, with a transition occurring around samples with a pore width of 2 nm. Owing to the complex solid–liquid interfacial coupling under extreme confinement below 2 nm, accurate prediction remains challenging. However, for systems with pore widths greater than 2 nm, the predictive model effectively offsets microscopic transient fluctuations through local statistical averaging, achieving high accuracy with mean relative errors of 0.042 and 0.051 for the overall and local predictions of pore water in the validation set, respectively, while improving computational speed by approximately three orders of magnitude.
(3) This study verifies the cross-scale and cross-structure generalization capability of the predictive model in rough pore systems. In a C-S-H model containing approximately 260,000 atoms and characterized by rough pore walls, the predictive model demonstrates excellent extrapolation capability across scales and structures. The study identifies 45 Å as the optimal local prediction size for this model, at which the relative error converges to its minimum value of 0.0736. While maintaining reliable accuracy, the predictive model improves computational efficiency by 3–4 orders of magnitude compared with conventional MD simulations. It not only provides a new route for the accurate and rapid evaluation of pore water diffusion within saturated C-S-H gel pores larger than 2 nm, but also lays a solid foundation for the future development of chloride ion diffusion prediction models in complex structures.
(4) Based on the modular computational framework, this study further looks ahead to a multiscale computational method. To overcome the limitation of fixed diffusion parameters in conventional numerical simulations, this method integrates atomistic structure-property prediction with mesoscale LBM. It enables bidirectional updating of the diffusion coefficient distribution and ion concentration distribution at each time step, thereby providing a new approach for more accurate simulation of mass transport in reinforced concrete.

The present study employs relatively fixed settings in terms of modeling parameters, structure construction methods, force field, and simulation workflows, and focuses exclusively on saturated pore systems at the nanoscale. Consequently, the current validation of the computational framework’s feasibility remains confined to these specified conditions. To expand the applicability boundaries of this framework, future work will systematically evaluate its transferability across diverse computational workflows and physicochemical conditions, further refine the predictive accuracy of the model, and progressively advance the development of a multiscale computational architecture.

## Figures and Tables

**Table 1:** Hyperparameter settings for the predictive model.

Parameter	Setting	Description
n_atom_basis	64	dimension of atomic feature vectors
cutoff	5 Å	cutoff radius
n_interactions	3	number of interaction layers
n_rbf	200	number of radial basis functions
hidden_layers	32	hidden layer size
activation function	ShiftedSoftplus	activation function type
batch	1	batch size (number of training samples per batch)
lr	5 × 10^−4^	initial learning rate with cosine annealing decay to 1 × 10^−6^ over total epochs
optimizer	ADAM	the choice of optimizer

## Data Availability

The original contributions presented in this study are included in the article/Supplementary Material. Further inquiries can be directed to the corresponding authors.

## Supplementary Materials

The following supporting information can be downloaded at: https://www.mdpi.com/article/10.3390/ma19122631/s1. Code S1. parametric modeling (Folder 1); Model S1. Partial examples of smooth pore models and bulk water model configurations (Folder 2); Code S2. Molecular dynamics (MD) simulation input files (Folder 3); Code S3. Preprocessing algorithms, database construction scripts, and dataloader implementation (Folder 4); Model S2. SchNet model (Folder 5); Code S4. Training script (Folder 6); Model S3. Rough pore model (Folder 7).

## Abbreviations

The following abbreviations are used in this manuscript:

C-S-H	calcium silicate hydrate
LBM	Lattice Boltzmann Method
NMR	nuclear magnetic resonance
QENS	quasi-elastic neutron scattering
DSC	differential scanning calorimetry
XTC	X-ray computed tomography
GNNs	graph neural networks
MD	Molecular dynamics
ML	Machine learning
DL	deep learning
AIMD	ab initio MD simulations
DFT	density functional theory
*MSD*	mean square displacement
MSE	Mean Squared Error
